# Supplementary material for: Long Non-Coding RNAs and Their “Discrete” Contribution to IBD and Johne’s Disease—What Stands out in the Current Picture? A Comprehensive Review
Source: Int J Mol Sci. 2023 Sep 1;24(17):13566. doi: 10.3390/ijms241713566 (PMC10487966; doi:10.3390/ijms241713566)
Supplement: Supplementary file 1 [file ijms-24-13566-s001.zip › ijms-2549260-supplementary.pdf]

Long Non-Coding RNAs and Their "Discrete" Contribution to IBD and Johne's Disease—What Stands Out in the Current Picture? A Comprehensive Review

Kostas A. Triantaphyllopoulos

Department of Biotechnology, School of Applied Biology and Biotechnology, Agricultural University of Athens, 75 Iera Odos St., 11855 Athens, Greece; ktrianta@aua.gr

TABLE OF CONTENTS **page**

Tables
Table S1.. 2
Table S2……………………………………… 4
Figures
Figure S1(A-B)...6
Figure S1(C-D)...8
ncFANdb 3.0 network input data……………...9

TABLES

Table S1. Expression Profile of known or novel, uncategorized RNAs in Crohn's disease, identified by Microarray GEO datasets (GSE75459, NCBI)

SEQUENCE_NAME	TRANSCRIPT_TYPE	log2(FC)	-log10(Pvalue)	ACCESSION	LENGTH (bp)	DESCRIPTION
**UPREGULATED**						
GUSBP 16, 3, 15,14	noncoding	9.291	11.749	NR_146391.1, NR_027386.2, NR_034021.1, NR_029426.1	2383, 1551, 1759, 1943	Homo sapiens GUSB pseudogene (GUSBP), non-coding RNA
TCONS_00021811, TCONS_00021810, TCONS_00021809, CONS_00021807, LOC100505918	noncoding	8.796	15.509	OA985557.1,OA985556.1,OA985555.1, OA985554.1, NR_037851.1	2195, 2291, 2214, 1897, 2442	Homo sapiens for PREDICTED lncRNAs (TCONS_00021811, TCONS_00021810, TCONS_00021809, TCONS_00021807) Homo sapiens uncharacterized LOC100505918, long non-coding RNA
LOC105375855,TCONS_00250763, TCONS_00250762	noncoding	8.011	3.438	XR_928920.2, OA989059.1, OA989058.1	7758, 1493, 2830	Homo sapiens uncharacterized LOC105375855 (LOC105375855), ncRNA Homo sapiens lncRNA for PREDICTED lncRNA (TCONS_00250763) Homo sapiens lncRNA for PREDICTED lncRNA (TCONS_00250762)
TCONS_00021999, TCONS_00021998, TCONS_00021997, GAS5-AS1	noncoding	7.888	12.07	OA985569.1, OA985568.1, OA985567.1, NR_037605.1	5143, 5706, 5182, 702	Homo sapiens lncRNA for PREDICTED lncRNA (TCONS_00021999) Homo sapiens lncRNA for PREDICTED lncRNA (TCONS_00021998) Homo sapiens lncRNA for PREDICTED lncRNA (TCONS_00021997) Homo sapiens GAS5 antisense RNA 1 (GAS5-AS1), LncRNA
FIGNL2-DT	noncoding	7.617	11.635	NR_135803.1	427	Homo sapiens FIGNL2 divergent transcript (FIGNL2-DT), LncRNA
**DOWNREGULATED**						
MK280060, lncAB107.3, LHRI_LNC1843.10, LHRI_LNC1843.3,LHRI_LNC1843.6, TALAM1, MALAT1 var3, 2 and 1	noncoding	-8.91	13.635	MK280060.1, MK280059.1, MN297067.1, MN297066.1, MN297065.1, NR_145459.1, NR_144568.1, NR_144567.1, NR_002819.4	6539, 3541, 3924, 4787, 4452, 8121, 8302, 8545, 8779	Homo sapiens gb|MK280060 lncRNA gene, complete sequence, Homo sapiens lncAB107.3 lncRNA gene, complete sequence, Homo sapiens LHRI_LNC1843.10 lncRNA gene, complete sequence, Homo sapiens LHRI_LNC1843.3 lncRNA gene, complete sequence,
ALOX12P2 var2, ALOX12P2 var1, ALOX12E	noncoding	-8.223	10.631	NR_120453.1, NR_002710.2, mRNA AF020774.1	2230, 2768, 2208	Homo sapiens arachidonate 12-lipoxygenase pseudogene 2 (ALOX12P2), transcript variant 2, ncRNA, Homo sapiens arachidonate 12-lipoxygenase pseudogene 2 (ALOX12P2), transcript variant 1, ncRNA, Homo sapiens hair and skin epidermal-type 12-

						lipoxygenase-relat
LOC440300, GOLGA2P8	noncoding	-7.705	13.386	NR_033738.1, NG_023539.1	7771, 7676	Homo sapiens chondroitin sulfate proteoglycan 4 pseudogene (LOC440300), non-coding RNA Homo sapiens GOLGA2 pseudogene 8 (GOLGA2P8) on chromosome 1
TRIM52 var2, TRIM52 var3, TRIM52 var1	noncoding	-7.432	13.399	NR_102760.1, NR_102761.1, NR_102759.1	851, 828, 1113	Homo sapiens TRIM52 antisense RNA 1 (head to head) (TRIM52-AS1), transcript variant 2, lncRNA, Homo sapiens TRIM52 antisense RNA 1 (head to head) (TRIM52-AS1), transcript variant 3, lncRNA, Homo sapiens TRIM52 antisense RNA 1 (head to head) (TRIM52-AS1)
TRIM52 var2, TRIM52 var3, TRIM52 var1	noncoding	-7.089	13.274	NR_102760.1, NR_102761.1, NR_102759.1	851, 828, 1113	Homo sapiens TRIM52 antisense RNA 1 (head to head) (TRIM52-AS1), transcript variant 2, lncRNA, Homo sapiens TRIM52 antisense RNA 1 (head to head) (TRIM52-AS1), transcript variant 3, lncRNA. Homo sapiens TRIM52 antisense RNA 1 (head to head) (TRIM52-AS1

Appendix: In light brown highlight are categorised the protein coding genes, without highlight ate shown the nonocoding transcripts, Downregulated, Upregulated, transcripts or genes in Crohn's disease versus normal (control group). **Platform GPL16956.** GSE75459 dataset, Agilent-045997 Arraystar human lncRNA microarray V3 (Probe Name Version) Agilent Technologies. The sequences and the names correspond to the order of the accession numbers, the accession lengths and the description in the table. Analysis was performed by GEO2R script (GEO, NCBI) as described in the text. The protein coding genes are highlighted in brown colour and the noncoding RNAs with no colour. log2(FC): Log2 fold change in a logarithmic scale of gene expression levels. -log10(Pvalue): log10 p value represents the level of significance of a gene or trait, showing the significance of fold-changes that deviate more strongly from zero. Protein coding RNAs (mRNAs) are not shown.

Table S2. Regulatory relationship between human lncRNAs and DNA methylation involved in Ulcerative Colitis.

TranscriptID	Transcript Name	Element type	Start	End	Disease Name	Resource	Technology	Status	Regulatory mechanism
ENST00000512369.1	DAPP1-002	5'UTR	99816846	99816913	Breast invasive carcinoma	TCGA	HM450k	hypomethylation	Cis-Methylated LncRNAs
ENST00000512369.1	DAPP1-002	5'UTR	99816846	99816913	Cervical squamous cell carcinoma and endocervical adenocarcinoma	TCGA	HM450k	hypomethylation	Cis-Methylated LncRNAs
ENST00000512369.1	DAPP1-002	5'UTR	99816846	99816913	Breast cancer	GSE60185	HM450k	hypomethylation	Cis-Methylated LncRNAs
ENST00000512369.1	DAPP1-002	5'UTR	99816846	99816913	Pancreatic ductal adenocarcinoma	GSE49149	HM450k	hypomethylation	Cis-Methylated LncRNAs
ENST00000512369.1	DAPP1-002	5'UTR	99816846	99816913	Prostate carcinoma	GSE34340	HM450k	hypermethylation	Cis-Methylated LncRNAs
ENST00000512369.1	DAPP1-002	5'UTR	99816846	99816913	Prostate cancer	GSE62053	HM450k	hypomethylation	Cis-Methylated LncRNAs
ENST00000512369.1	DAPP1-002	5'UTR	99816846	99816913	Ulcerative colitis	GSE32146	HM450k	hypomethylation	Cis-Methylated LncRNAs
ENST00000512369.1	DAPP1-002	5'UTR	99816846	99816913	Colon adenoma	GSE48684	HM450k	hypomethylation	Cis-Methylated LncRNAs
ENST00000512369.1	DAPP1-002	5'UTR	99816846	99816913	Bipolar disorder and iron deficiency (lung)	ENCODE	WGBS	hypermethylation	Cis-Methylated LncRNAs
ENST00000512369.1	DAPP1-002	5'UTR	99816846	99816913	Hepatocellular carcinoma	ENCODE	WGBS	hypomethylation	Cis-Methylated LncRNAs
ENST00000512369.1	DAPP1-002	5'UTR	99816846	99816913	Colon adenocarcinoma	TCGA	WGBS	hypomethylation	Cis-Methylated LncRNAs
ENST00000512369.1	DAPP1-002	5'UTR	99816846	99816913	Breast invasive carcinoma	TCGA	WGBS	hypomethylation	Cis-Methylated LncRNAs
ENST00000512369.1	DAPP1-002	5'UTR	99816846	99816913	Lung adenocarcinoma	TCGA	WGBS	hypomethylation	Cis-Methylated LncRNAs

ENST00000512369.1	DAPP1-002	5'UTR	99816846	99816913	Stomach adenocarcinoma	TCGA	WGBS	hypermethylation	Cis-Methylated LncRNAs
ENST00000296414.10	DAPP1-001	5'UTR	99816833	99816913	Ulcerative colitis	GSE32146	HM450k	hypomethylation	Cis-Methylated LncRNAs
ENST00000512369.1	DAPP1-002	5'UTR	99816846	99816913	Ulcerative colitis	GSE32146	HM450k	hypomethylation	Cis-Methylated LncRNAs
ENST00000296414.10	DAPP1-001	1_exon	99816833	99817014	Ulcerative colitis	GSE32146	HM450k	hypomethylation	Cis-Methylated LncRNAs
ENST00000512369.1	DAPP1-002	1_exon	99816846	99817014	Ulcerative colitis	GSE32146	HM450k	hypomethylation	Cis-Methylated LncRNAs
ENST00000296414.10	DAPP1-001	TSS200	99816633	99816832	Ulcerative colitis	GSE32146	HM450k	hypomethylation	Cis-Methylated LncRNAs
ENST00000512369.1	DAPP1-002	TSS200	99816646	99816845	Ulcerative colitis	GSE32146	HM450k	hypomethylation	Cis-Methylated LncRNAs
ENST00000534336.2	MALAT1	TERC promoter			Liver Cancer			hypomethylation	Trans-Methylation Due to LncRNAs
ENST00000534336.2	MALAT1	MALAT1 promoter			Non-Small Cell Lung Cancer			hypomethylation	Cis-Methylated LncRNAs

Note: TCGA: Cancer Genome Atlas, WGBS: Wide-genome Bisulfite sequencing, TSS: transcription start site. Evidence provided by: Lnc2Meth database, a manually curated database of regulatory relationships between lncRNAs and DNA methylation associated with human disease (see text). The methylation status of UC pathology is concerning the associated dual adaptor of phosphotyrosine and 3-phosphoinositides 1 (DAPP1) gene. DAPP1 is in genomic location: chr4:99,816,827-99,872,333, 4q23. Ensembl: ENSG00000070190, UniProt: Q9UN19. 5'UTR: 5' untranslated region, TSS200: gene region 200 bp downstream of transcription start site. 1_exon: 1st exon. The analysis platform used was HM450k Illumina Infinium 450k DNA methylation array, WGBS: whole-genome bisulfite sequencing.

FIGURES

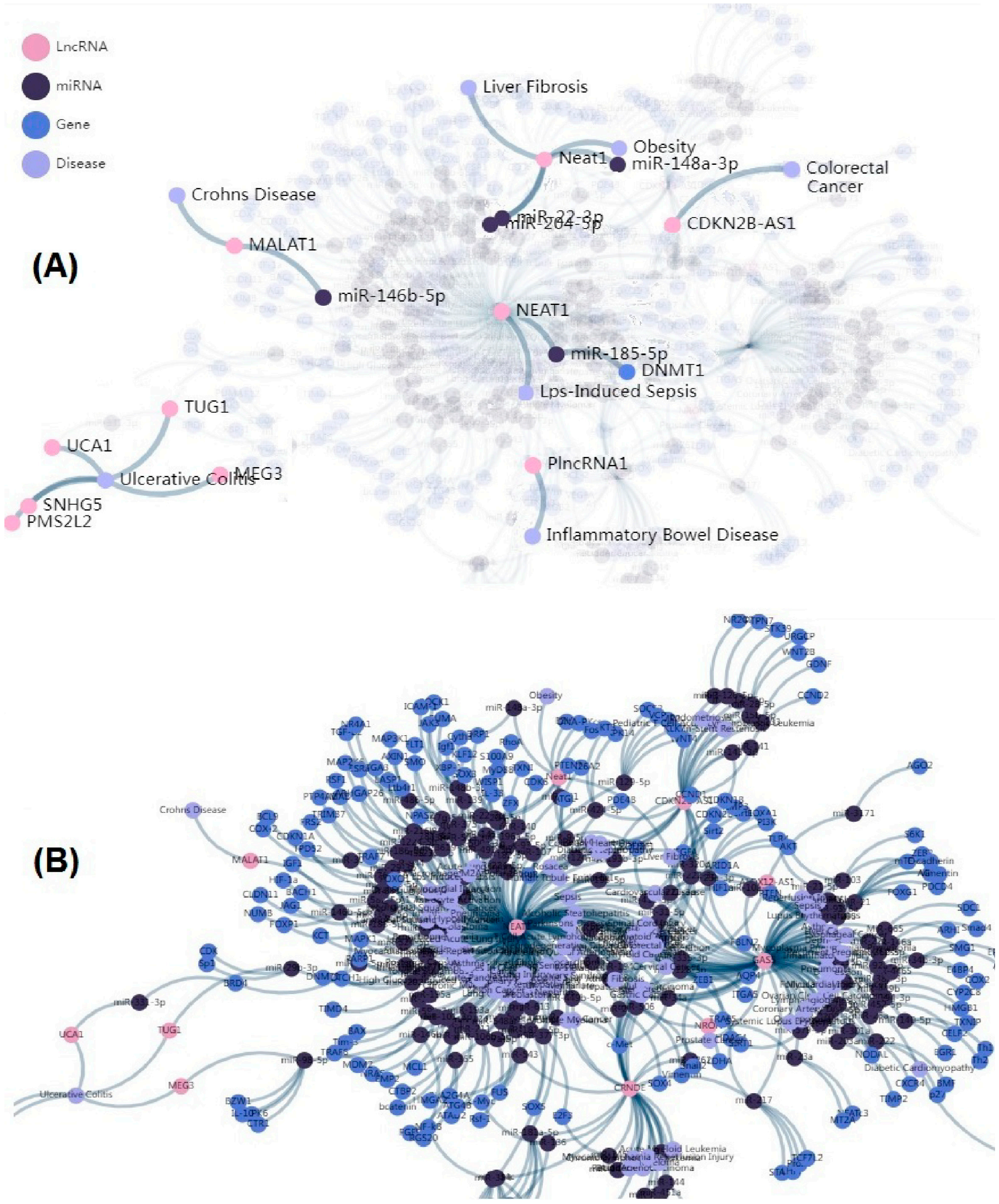
LncRNA
miRNA
Gene
Disease
(A)
Liver Fibrosis
Obesity
Neat1
miR-148a-3p
Colorectal Cancer
Crohns Disease
miR-22-3p
miR-204-5p
CDKN2B-AS1
MALAT1
miR-146b-5p
NEAT1
miR-185-5p
DNMT1
Lps-Induced Sepsis
TUG1
UCA1
MEG3
Ulcerative Colitis
PlncRNA1
SNHG5
PMS2L2
Inflammatory Bowel Disease
(B)

Figure S1. (A) LncRNA-gene-miRNA-disease network created in LncACTdb 3.0 database from human data, showing in this layer significant published targets for IBD in humans. (B) The full image shows the dense network with overlapping nodes, as calculated and visualized by the database visualization tool (details are described in the text).

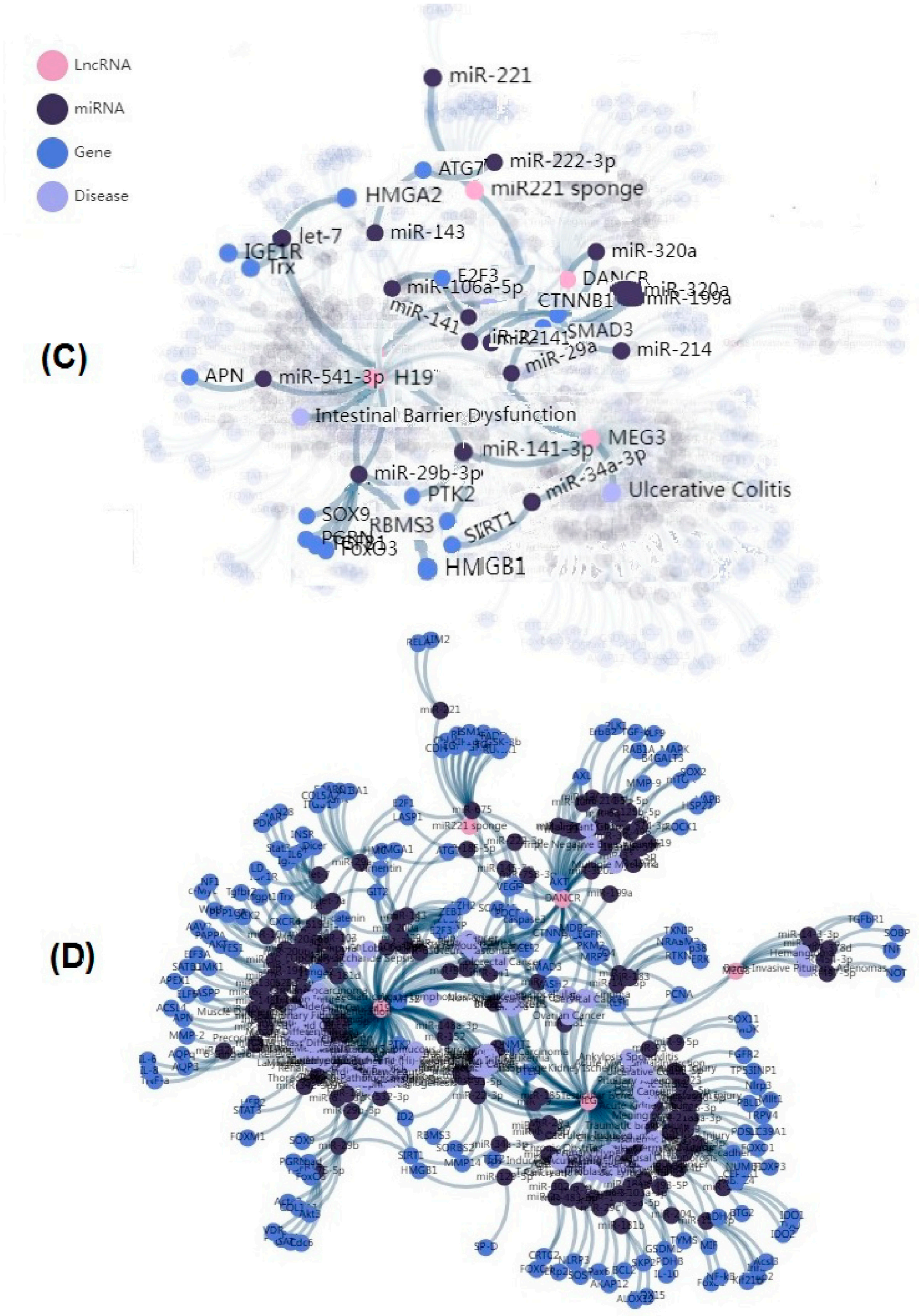

Figure S1. (C) LncRNA-gene-miRNA-disease network created in LncACTdb 3.0 database from cattle data, showing in this layer significant published targets for cattle infected with MAP. (D) The full image shows the dense network with the overlapping nodes, as calculated and visualized by the database (details are described in the text).

LncACT-Network tool settings and input genes used in LncACTdb 3.0 database.

Figure S1(A) and S1(B)

The input symbol/Ensembl IDs for the human IBD-related pathologies that were used in the LncACTdb 3.0 database for the analysis were the following:

lncRNA symbol/Ensembl ID: ALOX12-AS1, CDKN2B-AS1, CRNDE, GAS5, NRON, NEAT1
mRNA symbol/Ensembl ID: BAG4, GALNT10, EZH2, NFAT, STAT3
miRNAs: miR-495, miR-106b, miR-10b, miR-143-3p, miR-34a, let-7a
Diseases: Crohn's Disease, Inflammatory Bowel Disease, Ulcerative Colitis

Figure S 1(C) and S1(D)

The input symbol/Ensembl IDs for cattle infected with MAP used in the LncACTdb 3.0 database for the analysis were:

lncRNA symbol/Ensembl ID: ADNCR, DANCR, MEG3, MEG8, miR221 sponge, H19
mRNA symbol/Ensembl ID: YY1, Igf2, TSPAN3, ATXN7, Cdc42, ZFX
miRNAs: miR-204, miR-135b, miR-424, miR-127-3p, miR-136, miR-222
Diseases: Tuberculosis, Crohn's Disease
